# Explanations for failures in designed and evolved systems

**DOI:** 10.1093/pnasnexus/pgaf086

**Published:** 2025-04-02

**Authors:** Randolph M Nesse, Jay B Labov, Guru Madhavan

**Affiliations:** Center for Evolution and Medicine, Arizona State University, Tempe, AZ 85287, USA; Department of Psychiatry, University of Michigan, Ann Arbor, MI 48109, USA; National Academy of Engineering, Washington, DC 20001, USA; National Academy of Engineering, Washington, DC 20001, USA

**Keywords:** failure analysis, disease, engineering, evolutionary medicine, tacit creationism

## Abstract

Engineers have long studied the origins of design features that make machines prone to failure, but biologists have only recently begun investigating why organisms have traits that make them susceptible to disease. This article compares explanations for vulnerability to failure in machines with explanations for traits that make bodies vulnerable to disease. Some global explanations are relevant for both: design deficiencies, corrupted plans, assembly variations, incorrect operating environment, and trade-offs. These similarities suggest that a common framework for failure analysis could be valuable. However, a closer look at each of the 10 global categories reveals fundamental differences: machines are built to match an ideal blueprint, while species have no perfect genome or form. Design trade-offs in machines involve balancing multiple factors such as performance, robustness, and costs, while biological trade-offs maximize only gene transmission, often at the expense of health and lifespan. Detailed consideration of these and other differences reveals how the metaphor of body as a designed machine fosters tacit creationism that misrepresents the nature of organically complex systems.

## Introduction: failures in machines and bodies

Engineering and medicine both seek to understand the origins of failures in machines and bodies to prevent, repair, or cure them. Are the possible kinds of explanations for traits that make individual bodies and entire species vulnerable to disease the same as those for aspects of machines that makes them susceptible to failure? Finding the answer to this question is essential for 2 reasons. First, substantial similarities would suggest that a shared framework for failure analysis could enable valuable cross-pollination, with engineering approaches finding new medical applications and evolutionary insights informing engineering. Second, fundamental differences in explanations of failures in bodies and machines would reveal limitations in the metaphor of the body as a machine and the need for new approaches to understanding disease.

To set the scope of our analysis, we adopt some general definitions. A system can fail only if it can succeed, so failure is possible only for systems with a goal, purpose, aim, or function (1). “Failure” describes conditions ranging from inefficiency to total collapse. “Machines,” encompassing all human-designed products and systems, have functions ranging from opening cans to beaming back high-definition images from Mars. Their designs are products of engineering decisions that optimize trade-offs among multiple aims, including performance, robustness, efficiency, cost, longevity, and attractiveness. “Bodies,” describing all evolved living systems, are products of natural selection, which shapes systems that maximize gene transmission, often at the expense of health and longevity (2–4).

Engineers rely on several well-established frameworks to analyze failure, addressing both specific instances and systematic vulnerabilities (5, 6). These methods, including corrective and preventive actions, failure mode and effects analysis, and fault tree analysis, work synergistically to identify causes (7–11). Some approaches focus on quality control to prevent individual failures, while others examine design features that create vulnerability across entire product lines.

In medicine, explanations for disease have traditionally focused on why some individuals are susceptible, with less attention to traits that make all members of a species vulnerable, such as aging, the limited abilities to control infection and cancer, and the narrow human birth canal. Expanding the focus of explanation from the mechanisms that cause disease in individuals to traits that make all members of a species vulnerable launched the field of evolutionary medicine in the 1990s (12–20). Two broad categories of explanation for vulnerabilities are fundamental: the inherent limitations of natural selection and trade-offs that increase gene transmission at the expense of robustness.

The limitations of natural selection are well recognized. It cannot prevent all mutations, control all selfish genetic elements, or eliminate all developmental variations. As a stochastic process constrained by mutations, genetic drift, and path dependence, it cannot achieve or maintain perfectly optimal genotypes and phenotypes. It is slow to adapt long-lived organisms to environmental changes, and it is far too slow to keep pace with fast-evolving pathogens (14, 17, 21, 22). Some of these limitations parallel those explaining machine failures: design defects, faulty blueprints, assembly issues, or operation outside of environment specifications.

Trade-offs are as central to biology as they are to engineering, but different factors are involved. Engineered product designs often accept decreased robustness because of design trade-offs that enhance performance, cost savings, speed to market, or attractiveness. In contrast, traits shaped by natural selection are honed to maximize gene transmission, often at a cost to robustness (2, 23–27). Other traits give net survival and reproduction benefits to individuals that outweigh their risks to health (28). For instance, a strong immune response to diverse antigens comes at the risk of autoimmune and inflammatory diseases. Other traits, mainly those that maximize mating, harm individuals but may increase the probability of reproduction.

## Explanations for failures in engineered and evolved systems

Table 1 lists 10 possible explanations for failure that provide a framework for our analysis of the similarities and differences in engineering explanations for failures in machines and evolutionary explanations for aspects of bodies that make them vulnerable to diseases. Note that the aim is not to explain the sequence of events and causes for any individual instance of failure, but is instead to explain aspects of bodies and machines that make them vulnerable to failure.

**Table 1.** The origins of failures in engineered and biological systems.

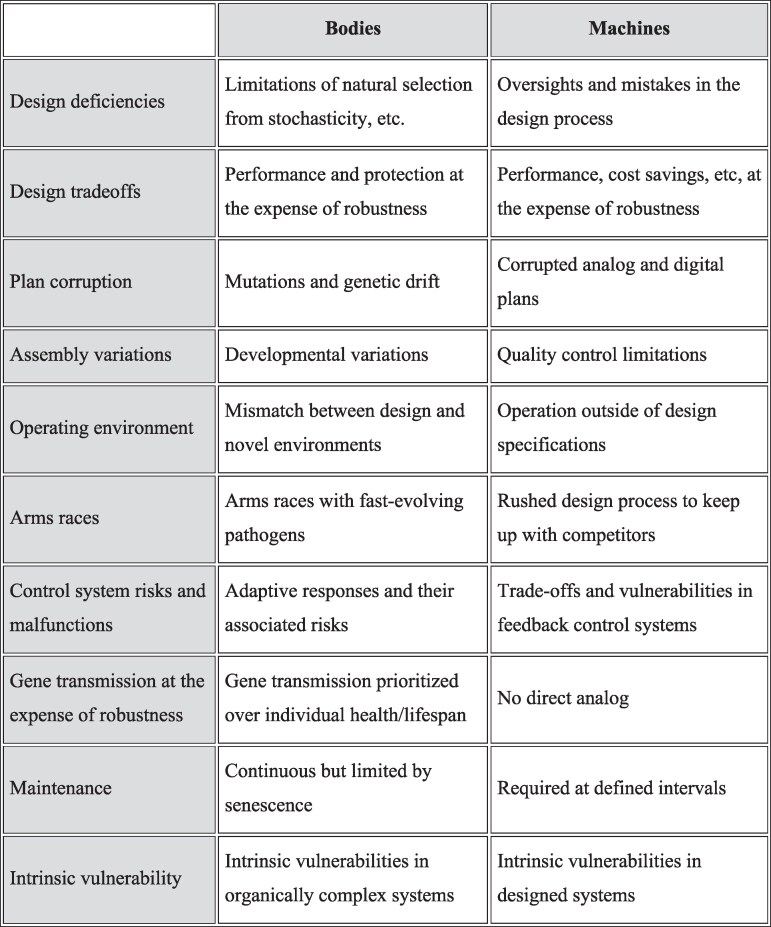

### Design deficiencies

Some aspects of a design are clearly deficiencies because more robust alternatives exist that would not compromise performance or increase costs. Such vulnerabilities have different origins in engineered and evolved systems.

In engineered systems, design deficiencies often stem from identifiable causes: technical oversights, production pressures, or other human factors. Some vulnerabilities arise from single oversights, like a bridge designed without considering wind vibrations (29). Others reveal deeper systemic issues. Analysis of the Boeing 737 Max 8's angle of attack sensor failure revealed a cascade of broader failures in design, review, reporting, and training procedures, ultimately traced to business incentives that compromised decision-making (30).

Biological design deficiencies, by contrast, reflect natural selection's inherent limitations. The birth canal's passage through the pelvis and the shared opening of windpipe and esophagus are compromised designs that persist because evolution cannot start fresh, it can only tinker with already existing structures. More robust alternatives may theoretically exist—an abdominal birth opening or separate air and food passages—but natural selection's constraints (stochastic processes, path dependence, limited strength, and genetic interactions) prevent their adoption.

The stochasticity of natural selection constrains living systems' optimality in ways foreign to engineered systems. Beneficial mutations may vanish if chance events eliminate their carriers. Genetic drift can make harmful mutations universal, especially in small or geographically isolated populations, or through hitchhiking by linkage with beneficial alleles on the same chromosome. Better designs may be theoretically possible but unreachable because the path requires temporarily reduced fitness or because necessary genetic variants never arise. A stark example is human vulnerability to scurvy—all humans are at risk because random mutations inactivated the gene for vitamin C synthesis (L-gluconolactone oxidase) millions of years ago when fruit-rich diets made the gene nonessential (31).

Path dependence—the inability to start fresh or revert to earlier stages—not only constrains bodies more severely, but also limits the robustness of machines. While radical redesigns are theoretically possible for machines, they are often limited by practical constraints of cost, time, established protocols, and compatibility requirements. Consider the persistence of outdated software in air traffic control systems, whose “fix on failure” approach led to the 2023 NOTAM system failure (32). However, biological path dependence is far more constrained—novel solutions like an abdominal birth canal or separate passages for air and food are extremely unlikely to be selected for, regardless of their potential benefits.

The limited strength of natural selection further compromises design optimality. Selection weakens at older ages when most population members have died from other causes, so genes that cause old-age diseases persist. Because deleterious recessive alleles are selected against only when they are paired with each other, conditions like cystic fibrosis or Tay-Sachs disease persist. A few diseases persist because individuals with 1 of each of 2 different alleles get advantages. Individuals with 1 regular and 1 sickle cell hemoglobin allele gain malaria protection, while homozygotes experience either sickle cell disease or higher risks of malaria (33). However, there are only a few other examples, most of which also protect against malaria.

Wrenching transitions to new environmental niches impose lasting costs to biological systems. Human upright posture led to knee pain, foot pain, hernias, hemorrhoids, varicose veins, fainting, and neck and back problems (34). Their persistence stems partly from natural selection's slowness and path dependence, and also because many traits result from multiple interacting genes affecting multiple functions. Similarly, in engineered systems, quickly adapting the Boeing 737 Max 8 to compete with new Airbus models created new risks. To mitigate the risk of stalling caused by moving the engines forward, angle-of-attack sensors were added to automatically push the airplane's nose down if a stall was imminent. However, the redesign omitted backup sensors, ignored warnings from engineers, and failed to adequately train all pilots to override the autopilot, so sensor failures caused nosedive crashes (35). Engineers can improve designs much faster than natural selection can—as evidenced by recent 737 Max models incorporating safety revisions.

Testing catches most engineering errors, and reports from servicing units identify more, allowing prompt fixes (including redesign) for problems. Natural selection, by contrast, is a constant process of testing of multiple variations, with those that survive and reproduce contributing more copies of code to future generations. The resulting mechanisms have structures different from those in machines. While machine parts typically serve discrete functions, body parts arise from multiple genes and often serve multiple networked functions, creating distinct patterns of robustness and fragility (36–40). Improving one trait can compromise others. For instance, strong selection for genes and traits that give advantages in the social-cultural niche may have interacted with other genes to disrupt brain development in ways that resulted in previously neutral variants increasing the risk of schizophrenia (41).

### Design trade-offs

In engineered systems, design trade-offs represent conscious compromises among many factors, but in biological systems, trade-offs compromise robustness in the service of one trait—increased gene transmission. These differences are fundamental, but there are also important similarities, and engineering methods have much to offer medicine (42). Engineers employ formal methods like Pareto optimization (describing situations in which improving one objective necessarily degrades others) to identify design frontiers. Some trade-offs are straightforward: lightweight race cars sacrifice durability for speed and battle tanks trade mobility for heavy armor. Other trade-offs require sophisticated quantitative modeling. In pharmaceutical manufacturing, designers plot drug purity against production speed, yield against energy costs, and batch size against quality control, seeking optimal compromises. As the business adage notes: cost, schedule, performance—pick any 2 (43).

Biological trade-offs, by contrast, emerge through implicit optimization without access to prescribed goals (26, 44, 45). Sweating protects against overheating while risking dehydration, and low blood pressure reduces cardiovascular disease risk while increasing fainting (46). Countless generations of selection pressure have shaped the immune system's balance between self-tolerance and pathogen recognition without explicit calculation of optima.

Less obvious biological trade-offs also provide important explanations for vulnerability (26, 47–50). Anxiety's false alarms are a cost of optimal protection against danger. Inflammation, with damage to normal tissue, is the price of infection control. A striking example comes from sheep on a North Sea island, where 47% of survivors through harsh winters show immune responses characteristic of lupus erythematosus—demonstrating how extreme conditions can reveal hidden trade-offs (51).

Future discounting represents a universal trade-off across both domains, but it operates differently in each. In biological systems, reproducing earlier in the lifespan increases fitness more than reproducing later because of mortality risks (52). This prioritization of immediate benefits is evident in aging—a prime contributor to human disease. While some aging tendencies arise from mutations that accumulated harmlessly when average lifespans were shorter in ancestral conditions, others, like inflammation, are selected for despite causing aging because they increase survival and reproduction early in life when selection is stronger (27, 53–56). From this perspective, health and longevity are fortunate side-effects of traits that were shaped to maximize gene transmission. Life history theory demonstrates how natural selection optimizes such trade-offs, as when organisms produce more numerous, smaller offspring sooner rather than fewer larger ones later (57, 58).

Engineering and economics parallel these biological trade-offs through time value calculations—cash now can be invested and earn interest, making current resources more valuable than future ones (59). Just as organisms sacrifice long-term health for immediate reproductive benefits, engineered systems often prioritize immediate market advantages over potential future riskier opportunities. Modern engineering systems increasingly blend these approaches through adaptive optimization, as seen in semiconductor fabrication lines that continuously adjust process parameters based on real-time measurements.

### Plan corruption

The idea that genomes are “blueprints” is widespread but misleading. Blueprints specify the ideal fixed design for a machine. Bodies have no single ideal type, but rather they are products of varying genes interacting with varying combinations of other genes in varying environments (60).

Some genetic variations are like a defect in a blueprint. A mutation that changes a single nucleotide can cause cystic fibrosis, and insertion of a string of repeating nucleotides causes Huntington's disease. However, hopes of finding specific genetic causes for common chronic diseases have given way to recognition that the risk of most diseases is influenced by complex interactions among many genes that each have only tiny effects (61). Environmental factors further exacerbate these vulnerabilities: The failure to close the neural tube during development can result in spina bifida, a defect influenced by genetic variations that is more common in environments where pregnant women may experience folic acid deficiency (62). Epigenetic modifications, such as DNA methylation, can also alter gene expression in response to environmental stimuli, creating lasting effects across generations that may not align with the gene's original function (63).

In engineering, blueprint copying errors are less of a problem now than corruption of computer code, and code corruption is less of a problem than programming errors, but all occur. The 1996 explosion of the Arianne rocket 5 seconds after launch resulted from reuse of code from Arianne 4 that could not convert a 64-bit floating point into a 16-bit signed integer (64). More recently, Log4J, a security flaw in the widely used Java library for logging data, allowed malicious actors to execute arbitrary code remotely. This flaw, stemming from inadequate validation of user input, was responsible for a critical exploit that affected millions of servers and applications worldwide. Its discovery in 2021 forced companies to issue emergency patches and underscored the challenges of maintaining security in increasingly interconnected software systems (65).

Errors arising from incomplete instructions can also cause catastrophic failures. For example, incomplete instructions for bolting door plugs in Boeing 737 Max 9 jets nearly caused an in-flight disaster (66). Engineering increasingly embraces flexibility in blueprinting through iterative design and modular systems. For example, agile software development incorporates continuous feedback to refine designs, mitigating risks from initial flaws. Similarly, parametric design in architecture allows blueprints to adapt to varying site conditions and material properties.

The contrast between designed and evolved systems is highlighted by how each system handles errors. Engineers can revise blueprints when flaws are discovered, though practical constraints often limit such corrections. In biological systems, genetic variations that decrease Darwinian fitness are purged only slowly and genetic variations that are detrimental in one context might provide advantages in another, making the concept of “error” more complex.

### Assembly variations

Development in biological systems is remarkably canalized but nonetheless vulnerable to variations resulting from stochasticity or the difficulty of controlling plasticity mechanisms that adapt the organism in response to signals about the current environment. For example, the developmental process of organogenesis involves precise timing and coordination among numerous molecular signals. Disruptions in these signals, whether due to genetic mutations or environmental exposures, can lead to congenital anomalies such as cleft palate or heart defects. Similarly, brain development—a highly complex process—is prone to subtle variations that can result in neurodevelopmental disorders like schizophrenia (36).

Engineered systems rely on precise, replicable assembly processes to ensure uniformity and reliability. However, even small deviations in these processes can have outsized impacts. For example, in semiconductor and communications equipment manufacturing, minor variations in environmental conditions can cause significant yield losses. To mitigate such risks, industries employ advanced quality control techniques, such as Motorola's pioneering Six Sigma initiative. Launched in 1986, it exemplifies engineered approaches to controlling variation and boosting reliability (67). Through extensive employee training and structured problem solving (define, measure, analyze, improve, and control, or DMAIC), Six Sigma has drastically reduced defects, saving billions of dollars (68).

### Operating environment

Both biological systems and engineered designs are challenged when operating outside their expected environments, but they respond to these challenges in crucially different ways. Biological systems often maintain partial function under stress thanks to distributed networked mechanisms, whereas engineered systems tend to fail completely when pushed beyond their design parameters.

There is no single ideal environment of evolutionary adaptiveness for humans (69), but modern conditions present many novel challenges. Natural selection is too slow to protect us from the consequences of abundant food, drugs, and leisure in developed societies. Preferences for salt, sugar, and fat, once advantageous on the African savannah, now drive epidemics of obesity, hypertension, and heart disease (21, 70, 71). This mismatch is exacerbated by cultural evolution: food products vary and selection by consumers with ancestral preferences interact with market forces to create processed foods, alcohol, and psychoactive drugs that overwhelm regulatory mechanisms. Other species face similar challenges when their evolved preferences lead them to remain in novel but suboptimal “ecological traps” (72).

Engineered systems, by contrast, are designed with precise operating parameters. A laptop's thermal management system, for example, works within specific temperature ranges, and aircraft materials are engineered to withstand calculated stress limits. Engineers can rapidly adapt designs to new conditions, such as modifying aerospace technologies for the harsh environments of Mars or adapting medical devices for extreme field conditions. For organisms, such changes can take thousands of generations in which many individuals are poorly adapted.

### Arms races

Pathogens evolve so rapidly that hosts struggle to keep pace. SARS-CoV-2 illustrates the challenge, evolving new variants faster than vaccine development cycles can adapt (73). The adaptive immune system, though extraordinary, comes at a high cost—acute immune responses can consume up to 25% of caloric expenditure (74). Additionally, the inflammatory response produces oxygen radicals that target pathogens but also damage normal cells (75, 76). The immune system must navigate a challenging optimization between self-tolerance and pathogen recognition (77, 78), which leaves organisms vulnerable to both infection and autoimmune disease (79, 80). Such costly risky defenses in evolved systems account for much disease, but comparable risks from defenses are necessary in engineered systems mainly in response to weapon competitions.

The closest things to “arms races” in everyday engineering arise from market competition and technological rivalry. The pressure to outpace competitors often results in rushed development cycles that compromise design integrity. Beyond the defects of Boeing's 737 Max, prominent examples include the premature release of the Ford Edsel (1957), the bug-ridden launch of Windows Vista (2006), and the missteps of Google's “smart glasses” (2014).

Biological systems evolve with no capacity for “foresight.” Host-pathogen interactions, for example, never reach equilibrium; any temporary advantage for the host is quickly countered by pathogen adaptations. Conversely, goal-defined engineered systems can adapt to prevent failures through systematic iterations and improvements. Collaborative efforts, such as international standards and industry agreements, can stabilize technological development in ways that are unimaginable in biological contexts.

The results of such differences become clear when examining specific systems. For example, kidneys excel at filtering multiple substances simultaneously, adapting fluid and solute balance to varying conditions. However, industrial filtration achieves far higher throughput and specificity using engineered membranes under tightly controlled conditions. Similarly, photosynthesis, though elegant, typically converts only about 1% to 2% of solar energy into biomass—significantly less than the 15% to 20% efficiency achieved by commercial solar cells (81, 82). However, this simple comparison does not tell the whole story, as photosynthesis also produces complex organic molecules and self-repairing systems, while solar cells produce only electricity.

### Control systems

In engineered systems, control often aims for precise calibration. The proportional–integral–derivative controller exemplifies this approach, calculating corrections based on current error (proportional), accumulated error (integral), and rate of change (derivative). These calibration systems excel at maintaining tried-and-tested setpoints but can fail consequentially outside design parameters. Signal detection theory guides the optimization of detection systems (83), balancing false alarms against missed detections in applications from automated braking to fire suppression systems.

Biological control systems regulate defensive responses—such as blinking, shivering, sweating, and panic—through mechanisms that reflect evolutionary cost-benefit ratios (48, 84, 85). In life-threatening situations, the cost of a false alarm is small compared to the cost of failing to respond to a threat, so optimal systems tend to produce false alarms. This “smoke detector principle” explains apparent overreaction to threats in anxiety disorders and it is essential for making good medical decisions about blocking defensive responses like fever, pain, vomiting, or inflammation (86).

Control systems in both domains are intrinsically vulnerable to instability and positive feedback (48, 85, 87–89). In engineered systems, sprinkler activation errors exemplify the risk—leaks or discharges provoked by very small fires can result in substantial water damage. In biological systems, pain and anxiety disorders emerge when systems meant to adjust protective responses become overly sensitized. Repeated danger exposure can create vicious cycles where fear breeds more fear (90). Depression's “kindling” phenomenon, in which episodes increase future vulnerability, may reflect a control system adapting to an environment in which effort consistently fails (91).

The architecture of control differs fundamentally between domains. Engineered systems ideally employ hierarchical control with clear command structures and redundancy through identical parallel systems. Blood pressure regulation illustrates the biological alternative—multiple interacting systems (baroreceptors, hormones, kidneys, autonomic responses) provide partial, overlapping compensation. This distributed architecture enables graceful degradation rather than catastrophic failure.

Control system malfunction manifests in disease through multiple pathways (48, 88) High baseline blood pressure leads to hypertension; glucose regulation failures produce “brittle diabetes” that cause severe swings in blood glucose levels; bipolar disorder shows a pattern of oscillation and bistability that typically results from excessive gain. Cancer represents perhaps the ultimate control failure, in which cells that escape regulation reproduce with accumulating errors that take them further outside of control systems (92–95).

### Gene transmission at the cost of robustness

While both domains involve trade-offs between performance and durability, only biological systems are shaped by selection pressures that routinely sacrifice individuals' survival for reproductive success. Traits that increase reproduction often directly compromise individual health and survival (96–100). Male peacocks' elaborate tails attract mates but impair flying ability; stags' massive antlers aid mate competition but demand such energy that they are shed annually after mating and then regrown. In primates, testosterone-driven immune suppression and risk-taking help explain males' shorter lifespans (101–103).

Machines exhibit superficially similar trade-offs—sports cars sacrifice efficiency for performance, smartphones prioritize aesthetics over repairability—but these serve human-defined goals rather than reproductive imperatives. While Formula 1 engines operate at mechanical limits and consumer electronics embrace planned obsolescence, these represent conscious design choices rather than inevitable selective pressures.

The contrast extends to altruistic behaviors. In biological systems, traits that decrease individual reproductive success may be selected for if they sufficiently benefit genetic relatives (104–106). Such “kin selection” favors tendencies to extreme parental sacrifice because offspring share 50% of parental genes. While engineered systems might be programmed for self-sacrifice—like swarm robots protecting higher-value units or military drones accepting damage to protect assets—these represent programmed behaviors serving human objectives rather than emergent properties shaped by reproductive success.

Product ecosystems offer the closest engineering parallel, in which companies might sacrifice individual product longevity for brand survival. Apple's frequent iPhone iterations, disposable fast fashion, and rapid software update cycles prioritize market success over product durability. Yet, even these examples highlight the fundamental difference: engineered trade-offs serve defined metrics of market success, while biological trade-offs emerge from blind selection for gene transmission, often at significant cost to individual health and survival.

### Maintenance

Engineered and evolved systems approach repair and renewal in fundamentally different ways. While engineered systems require scheduled external maintenance that often falls victim to future discounting, biological systems employ continuous self-repair that ultimately faces evolutionary constraints.

In engineered systems, maintenance requirements are specific and critical (9). Infrastructure like bridges and power plants demand rigorous scheduled inspections; aircraft require mandatory component replacement at specific intervals; industrial equipment increasingly uses predictive analytics to anticipate failures. Nuclear power plant engineers must manage complex interactions between material degradation, changing regulatory requirements, and shifting safety standards. Maintaining these systems requires knowledge preservation across decades, often outlasting the careers of original designers—a fundamental difference from biological maintenance mechanisms.

Yet, future discounting—in which present costs outweigh future benefits—often leads to maintenance deferral. Even simple maintenance like automotive oil changes, though cost-effective, are often neglected until damage occurs. The 2021 catastrophic Surfside, Florida, condominium collapse catastrophically demonstrates this principle.

Biological systems, in contrast, perform continuous self-maintenance and have theoretically unlimited capacity for repair and replacement—lizards regenerate tails, and organ systems constantly renew themselves. Sleep exemplifies biological maintenance—a daily investment in repair that evolution has preserved despite vulnerability during unconsciousness. However, evolution rarely selects for extended regenerative capabilities because limited lifespans in natural environments make future discounting a powerful barrier to longevity investments (107). Natural selection favors early reproduction over long-term durability, leading to limited investment in repair systems that might extend lifespan beyond the age at which an individual cannot reproduce or increase the reproduction of kin.

The manifestation of future discounting differs fundamentally between domains. In engineered systems, specific failure patterns appear; buildings deteriorate until reaching critical points, machinery breaks down when routine service is skipped, software accumulates “technical debt” in which quick fixes create mounting challenges, and transportation systems face cascading failures from maintenance backlogs. Each represents a conscious (though often flawed) decision to defer present costs despite greater future consequences.

Modern engineering increasingly adopts biological insights through predictive maintenance and self-healing materials. However, even these remain fundamentally different from biological maintenance—they represent programmed responses to anticipated failures rather than emergent properties shaped by evolutionary pressures. The key distinction lies not in the maintenance mechanisms but in the forces shaping them: conscious design versus blind selection.

### Intrinsic vulnerabilities

The laws of physics, chemistry, and control systems make some engineered and evolved systems vulnerable to failure no matter how they are designed. Any closed space with a small opening is vulnerable to explosion if the outlet is obstructed, so the appendix and rocket boosters are prone to rupture. Control systems are vulnerable to oscillation and runaway positive feedback loops. Nuclear reactors are inherently vulnerable to positive feedback failures, and bodies are vulnerable to cytokine storms.

The approaches to understanding and managing intrinsically vulnerable systems reveal fundamental differences between domains. Engineering emphasizes systematic documentation and analysis, particularly in safety-critical systems. Industrial robotics development exemplifies this—each accident triggers specific design modifications, new safety protocols, and industry-wide sharing of lessons learned. Nuclear facilities maintain detailed failure logs and require root cause analysis of even minor incidents. Aviation safety evolves through rigorous investigation of every accident and near miss.

While engineers can modify designs based on documented failures, evolution works via a process of massive parallel experimentation, testing countless variations simultaneously across populations. Biological systems develop redundant, overlapping functions that degrade gracefully under stress rather than failing catastrophically. This approach contrasts sharply with engineered systems' typically more linear failure modes and documented response protocols.

## Limitations

This analysis focuses on design deficiencies and other factors that influence all members of a species or product line. Strategies for investigating specific instances of failure are a crucial source of information about system vulnerabilities but they are not covered here. We chose 10 categories of possible explanations for failure vulnerabilities, but choosing more, fewer, or different categories could give different results. Our analysis does not claim to have immediate practical value, but its descriptions of the major similarities and differences between the origins of failure in bodies and machines are likely to be useful. Also, the article does not consider the role of social processes in the origins of engineering design deficiencies; this important source of failures will be the topic of future work.

## Conclusions and future directions

This report is the first attempt to systematically analyze the similarities and differences in explanations for failures in bodies versus those in machines. We hoped to find similarities that could provide a framework for more systematic failure analyses and differences whose neglect may be obstructing progress in understanding disease. We found both, but the similarities were overwhelmed by the substantial differences.

The similarities are nonetheless substantial, and the general framework that they provide may apply to functional systems in general. Whether designed or evolved, systems can fail because of suboptimal design, assembly variations, operation in novel environments, trade-offs, and 6 other possible explanations. They provide a rough framework and theoretical foundation for organizing possible causes of failure. Although they are quite general, considering each of them in turn could make failure analysis more systematic.

We had hoped that the categories of failure analysis from biology would have direct applications in engineering, but their utility remains uncertain. For instance, while path dependence is an important explanation for bodily vulnerabilities, engineers are already aware of the costs and benefits of starting from scratch to correct a design that is prone to failure. Selection that maximizes gene transmission at the expense of health is one of several possible origins of failure in bodies that do not have direct analogs in engineering. However, biomimicry is well established in bioengineering, and evolutionary methods are proving useful in synthetic biology (108–110). Engineers who grasp new evolutionary ideas about the origins of failures may well see applications that we do not.

Failure analysis strategies from engineering have underappreciated applications in biology. For instance, analyses of trade-offs that compromise robustness have long been routine in engineering but their role in explaining disease vulnerability is only now being recognized. Examples include cancer as a price for tissue repair, and autoimmune disease as the price of an effective immune system. The role of control system malfunctions in disease is recognized, but the sophisticated analytical methods that are routine in engineering have only begun to be applied in medicine. While some authors have worked at this interface, opportunities abound for progress from transdisciplinary collaborations (42, 48, 88).

Recognizing the fundamental differences between the origins of failures in biological and engineered systems is more consequential than recognizing their similarities. The pathways to vulnerabilities for bodies and machines are quite different for each of the 10 categories. Engineering design deficiencies are products of limited time and expertise, while bodily design deficiencies result from stochasticity, path dependence, and how pleiotropy and epistasis give rise to organically complex systems. Blueprint corruption can be minimized, but organisms have no ideal type, genetic drift is inherent, and mutations are recurrent and only slowly purged. Manufacturing variations can be minimized by quality control protocols, but organismal development cannot be completely canalized, and adaptive plasticity in response to environmental cues is inherently vulnerable to errors. Operation outside of specified environments causes machine failures, but redesign can improve reliability in extreme environments. Bodies also falter in environments unlike those they evolved in—especially modern ones designed to satisfy human desires, often at great cost to health. Corporate competition that can compromise design optimality is gentle compared with the constant competition that organisms face from pathogens and the risks and costs of defensive systems. Control systems in machines and organisms follow the same principles, but the structure of organically complex control systems is fundamentally different from that in designed systems. Robustness is one of many design considerations for engineers, but organisms are shaped to maximize gene transmission at the expense of robustness and all other benefits. Machines need regular maintenance that usually requires downtime, but bodies are maintained continually. Finally, both bodies and machines have intrinsic vulnerabilities arising from the laws of chemistry and physics; however, the structure of complexity in organic systems makes them vulnerable and robust in ways different from machines.

These differences are vastly underappreciated thanks to the pervasiveness of the metaphor of body as machine. The metaphor has been useful to counter mystical notions that life involves some special substance or principle that is outside the realm of physical mechanisms, but its literal application promotes a distorted view of organic systems. A critique by Richard Lewontin notes that “[T]he ur-metaphor of all modern science, the machine model that we owe to Descartes, has ceased to be a metaphor and has become the unquestioned reality: Organisms are no longer like machines, they are machines” (111). His critique joins many articles that document the distortions promoted by the literal interpretation of the metaphor (112–115). Those distortions reflect a tendency to “tacit creationism” that disavows a designer but nonetheless views bodies as if they were products of conscious design, assuming that they are composed of distinct parts with specific functions connected in simple networks and that failures result from specific flaws in specific parts (116). Many diseases do arise from specific causes, but most common chronic diseases result from the small effects of many genes interacting with other genes and varying environments. Asking why natural selection leaves some systems vulnerable, and considering all 10 possible explanations, offers a new perspective on such diseases.

Considering the different origins of failures calls special attention to the different structures of complexity in designed and evolved systems. The parts of a machine tend to have sharp boundaries, specific functions, and a few connections to other parts. Robustness is a product of good design that may include backup systems that kick in when a failure is detected. Many parts of a body have indistinct boundaries, multiple functions, and networked connections to many other parts. This is especially relevant for genetics. Students start by learning about Mendelian traits that are influenced by single genes, giving the impression that most genes have single specific functions. Physiology continues the tendency by describing *the* function of molecules like serotonin (it has dozens), and anatomy does the same by attributing a specific function to brain regions like the amygdala (it too serves multiple functions). Studies of how genes influence disease risks tend to view specific alleles as “genes for” disease x or y, despite the vast data showing that the risks for most diseases are influenced by the tiny effects of thousands of alleles that also influence the risk of other diseases.

The metaphor of body as machine will persist because describing the body's systems as mechanisms is so useful. Avoiding the associated distortions will require recognizing that the complexity in organic systems is not just greater than that in designed systems, but also fundamentally different. Nowhere is this more obvious than in considering how explanations for failure differ in evolved and designed systems.

This perspective article calls attention to the opportunities at the intersection of engineering, evolutionary biology, and medicine, but it hardly begins to explore those opportunities. That will require finding support for collaborative efforts by specialists with very different areas of expertise. Our tentative conclusion that engineering expertise offers greater potential for understanding disease than biological explanations offer for understanding engineering failures may turn out to underestimate the ability of engineers to find new creative applications. Just as evolutionary medicine helped transform understanding of the ultimate causes of disease, comparative failure analysis could yield novel insights for both domains. Engineers may draw inspiration from biology's distributed, adaptive architectures, while biologists might benefit from engineering's rigorous failure analysis methodologies. Breakthroughs will likely emerge not from direct imitation, but rather from abstracting and adapting fundamental underlying principles.

We conclude with recognition that efforts to transcend disciplinary boundaries are intrinsically vulnerable to failure. Understanding the origins of that risk can increase the success of efforts to better understand the shared and different origins of failures in bodies and machines, and more importantly, how such understanding can prevent failures.

## Data Availability

There are no data underlying this work.
